# Supplementary material for: Deletion of a Csf1r enhancer selectively impacts CSF1R expression and development of tissue macrophage populations
Source: Nat Commun. 2019 Jul 19;10:3215. doi: 10.1038/s41467-019-11053-8 (PMC6642117; doi:10.1038/s41467-019-11053-8)
Supplement: Supplementary file 1 — Supplementary Information [file 41467_2019_11053_MOESM1_ESM.pdf]

Deletion of a *Csf1r* enhancer selectively impacts CSF1R expression and development of tissue macrophage populations.

Rojo et al.

Contents

Supplementary Figures 2

Supplementary Figure 1 – Validation of guide RNAs targeting FIRE. 2

Supplementary Figure 2 – Deletion of FIRE had no effect on intestine morphology or numbers of Paneth and Goblet cells. 3

Supplementary Figure 3 – Deletion of FIRE did not affect myeloid cells or their progenitors in bone marrow but reduced their differentiation in the presence of CSF1. 4

Supplementary Figure 4 – Deletion of FIRE had no effect on macrophage populations in the liver or spleen. 6

Supplementary Figure 5 – Deletion of FIRE had no effect on macrophage populations in the lung. 8

Supplementary Figure 6 – Deletion of FIRE had no effect on macrophage populations in the intestines. 9

Supplementary Figure 7 – Flow cytometry gating strategies for peritoneal cavity, heart and kidney. 11

Supplementary Figure 8 – Further analysis of *Csf1r* $^{\Delta FIRE/\Delta FIRE}$ brains 13

Supplementary Tables 15

Supplementary Table 1– Oligonucleotides used for sequencing FIRE deletions 15

Supplementary Table 2– Flow cytometry antibodies 16

Supplementary Table 3 Primers used in qRT-PCR 17

References 18

Supplementary Figures

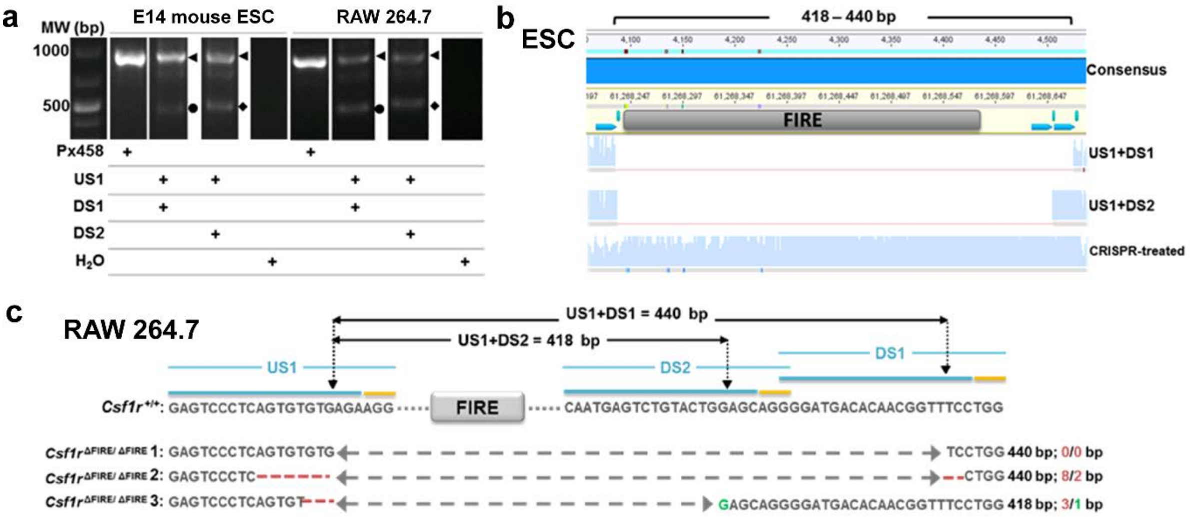

Supplementary Figure 1 – Validation of guide RNAs targeting FIRE.

a, DNA-targeting efficiency of guide RNA (gRNA) pairs in pools of EGFP$^+$ E14 mouse embryonic stem cells (ESC) and RAW 264.7 cells. Representative gel images show PCR products. Px458 = no gRNA control, arrows = wild-type allele, circles = mutant allele (US1+DS1), diamonds = mutant allele (US1+DS2), MW = molecular weight, bp = base pair. **b,** Representative analysis of Sanger sequencing results using Geneious version R8[1]. From top to bottom, the first and second chromatograms show the fragment of DNA deleted in *Csf1r*$^{\Delta FIRE/\Delta FIRE}$ E14 ESC clones. The third chromatogram shows the presence of FIRE in CRISPR-treated *Csf1r* $^{+/+}$ clones. **c,** Schematic of the FIRE element in CRISPR-treated RAW 264.7 cells showing the location of gRNAs (blue lines) and PAM sequences (yellow lines). *Csf1r*$^{+/+}$ clones had no mismatches, relative to the reference FIRE sequence. All *Csf1r*$^{\Delta FIRE/\Delta FIRE}$ clones had slightly different deletions at the endogenous FIRE sequence; red-dashed lines and green text represent additional deletions and insertions, respectively. The number of modified bp upstream or downstream the FIRE sequence is specified at the left or right side of the slash symbol and is color-coded. All source data are provided within a Source Data excel file.

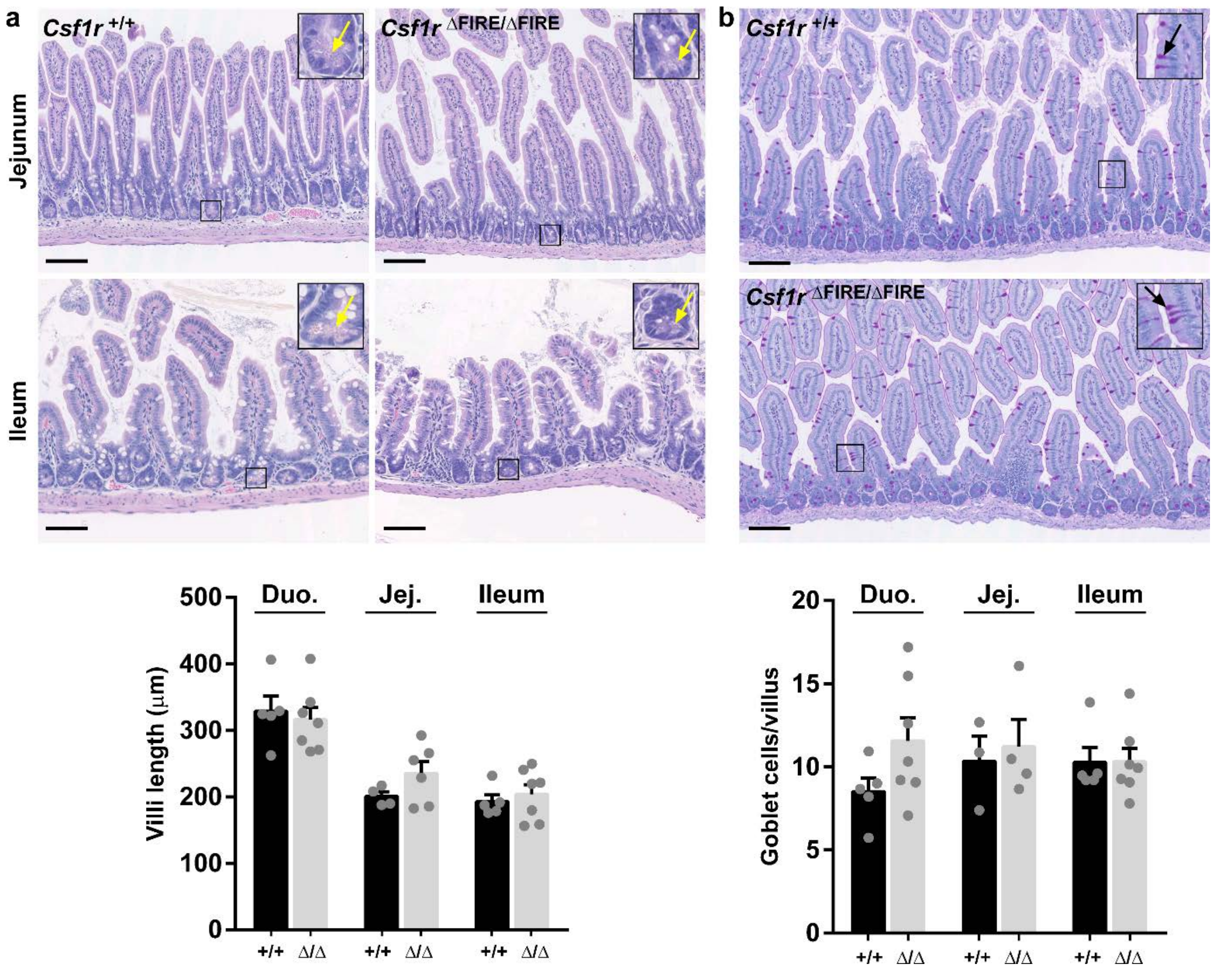

Supplementary Figure 2 – Deletion of FIRE had no effect on intestine morphology or numbers of Paneth and Goblet cells.

a, Formalin-fixed and paraffin-embedded (FFPE) jejunum (Jej.) and ileum sections from the small intestines of 10-13 week old mice of each genotype were stained with Hematoxylin and Eosin. Yellow arrows point to Paneth cells. Scale bars = 100 μm. Villus length was calculated using NDP.view2 software. n = (Duo., Jej., Ileum) = 5, 4, 5 +/+ and 7, 6, 7 Δ/Δ mice. **b,** FFPE duodenums (Duo.) from 12-week old mice were stained for periodic acid-schiff (PAS). Black arrows point to Goblet cells. Scale bars = 100 μm. The number of Goblet cells per villus was calculated using ImageJ. n = (Duo., Jej., Ileum) = 3, 5, 5 +/+ and 7, 4, 7 Δ/Δ mice. All source data are provided within a Source Data excel file. Graphs show mean + SEM and *P* values were determined by two-tailed t-tests.

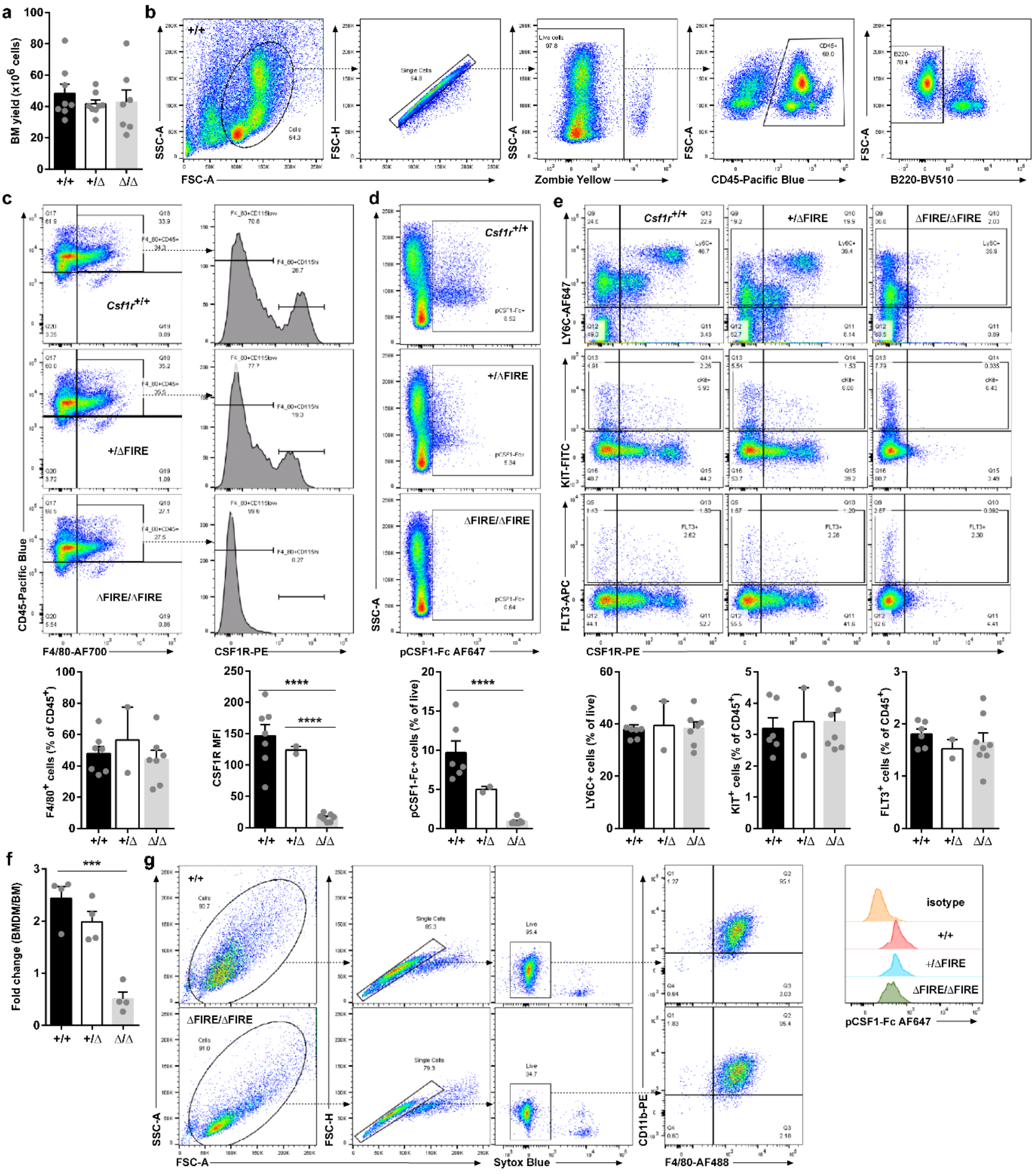

Supplementary Figure 3 – Deletion of FIRE did not affect myeloid cells or their progenitors in bone marrow but reduced their differentiation in the presence of CSF1.

a, Bone marrow (BM) cells were isolated and counted from mice aged 12-13 weeks. n = 8 (+/+, +/Δ) and 7 (Δ/Δ) mice from 4 experiments. **b,** BM gating strategy for flow cytometry analysis. **c,** BM cells were isolated from mice aged between 8-10 weeks and analyzed by flow cytometry. CD45$^+$F4/80$^+$ cells were examined for surface CSF1R expression, calculated as median fluorescence intensity (MFI). P < 0.0001 (****). **d,** BM cells from the same cohort of mice above were analyzed by flow cytometry for binding of pCSF1-Fc^{AF647}. P < 0.0001 (****). **e,** Live BM cells from the same cohort of mice above were analyzed by flow cytometry for

LY6C expression. $CD45^{+}B220^{-}$ cells were analyzed for FLT3 and KIT expression. **f,** BM cells were isolated from mice aged 12-13 weeks and cultured in recombinant human CSF1 for 7 days to produce BM-derived macrophages (BMDM). Graph shows the fold change of BMDM produced/seeded BM. *P* = 0.0003 (***). **g,** BMDM from the same cohort of mice above were analyzed by flow cytometry for expression of F4/80, CD11b and binding of pCSF1-Fc^{AF647}. The histogram shows typical pCSF1-Fc^{AF647} binding to the BMDM generated from each genotype. All source data are provided within a Source Data excel file. Graphs show mean + SEM and *P* values were determined by two-tailed t-tests.

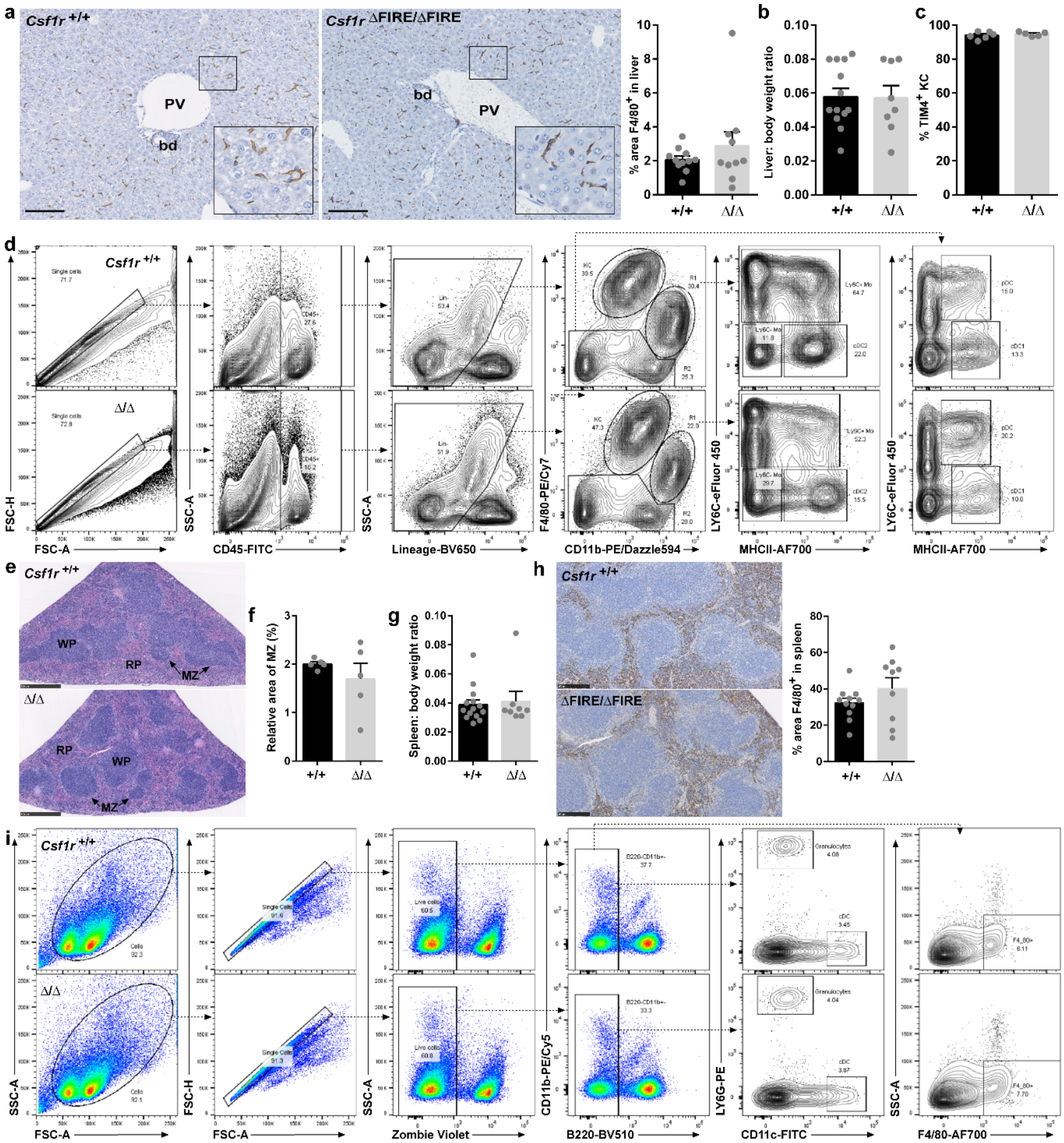

Supplementary Figure 4 – Deletion of FIRE had no effect on macrophage populations in the liver or spleen.

a, Formalin-fixed and paraffin-embedded (FFPE) livers from 3 – 7 week old *Csf1r*$^{\Delta FIRE/\Delta FIRE}$ mice (Δ/Δ) and controls (+/+) were stained with an antibody against F4/80. PV = portal vein, bd = bile duct. Scale bars = 100 µm. The percentage area of F4/80 staining was calculated using ImageJ. n = 11 +/+ and 10 Δ/Δ. **b,** Liver to body weight ratios were determined from mice aged between 6 – 12 weeks. n = 14 +/+ and 8 Δ/Δ. **c,** Single cell suspensions of digested livers from mice aged between 8-10 weeks were analyzed by flow cytometry. Kupffer cells (KC) were gated as $CD45^{+}$, lineage^{-} (Lin^{-}; $CD3^{-}CD19^{-}LY6G^{-}$) and $F4/80^{hi}CD11b^{+}$ to analyze TIM4 expression. n = 6 +/+ and 5 Δ/Δ from 3 repeat experiments. **d,** Leukocyte populations

were isolated from enzymatically digested livers. The flow cytometry profiles show analysis of representative *Csf1r*$^{+/+}$ and *Csf1r*$^{\Delta FIRE/\Delta FIRE}$ mice using the gating strategy described in [2] and presented in Fig.4a. Monocyte/macrophages were identified as CD45^{+} (Panel 2) lineage-negative (lacking lymphocyte and granulocyte markers (CD3, CD19 and LY6G), (Panel 3). KC were identified as F4/80^{hi}CD11b^{lo} (Panel 4). F4/80^{lo}CD11b^{hi} and F4/80^{-}, CD11b^{-} populations were further separated on the basis of Ly6C and MHCII expression (Panels 5 and 6). **e,** FFPE spleens from 4 – 7 week old *Csf1r*$^{\Delta FIRE/\Delta FIRE}$ mice (Δ/Δ) and controls (+/+) were stained with Hematoxylin and Eosin. WP = white pulp, RP = red pulp, MZ = marginal zone. Scale bar = 500 µm. **f,** The area occupied by the MZ, relative to the whole spleen area was quantified using ImageJ. n= 5 mice per genotype. **g,** Spleen to body weight ratios were determined from mice aged between 6 – 12 weeks. n = 14 +/+ and 8 Δ/Δ. **h,** FFPE spleens from 3 – 7 week old *Csf1r*$^{\Delta FIRE/\Delta FIRE}$ mice (Δ/Δ) and controls (+/+) were stained with an antibody against F4/80. The percentage area of F4/80 staining was calculated using ImageJ. n = 11 +/+ and 9 Δ/Δ. **i,** Leukocyte populations were isolated from enzymatically digested spleens. The flow cytometry profiles show the full analysis of representative *Csf1r*$^{+/+}$ and *Csf1r*$^{\Delta FIRE/\Delta FIRE}$ mice shown in Fig.4b. Granulocytes were identified as CD11b^{+}B220^{-} (Panel 4) and CD11c^{-}LY6G^{+} (Panel 5). Conventional dendritic cells (cDC) were identified as CD11b^{+}B220^{-} (Panel 4) and CD11c^{+}LY6G^{-} (Panel 5). Red pulp macrophages were identified as CD11b^{+}B220^{-} (Panel 4) and F4/80^{+}SSC^{lo} to exclude granulocytes (Panel 6). All source data are provided within a Source Data excel file. Graphs show mean + SEM and *P* values were determined by two-tailed t-tests.

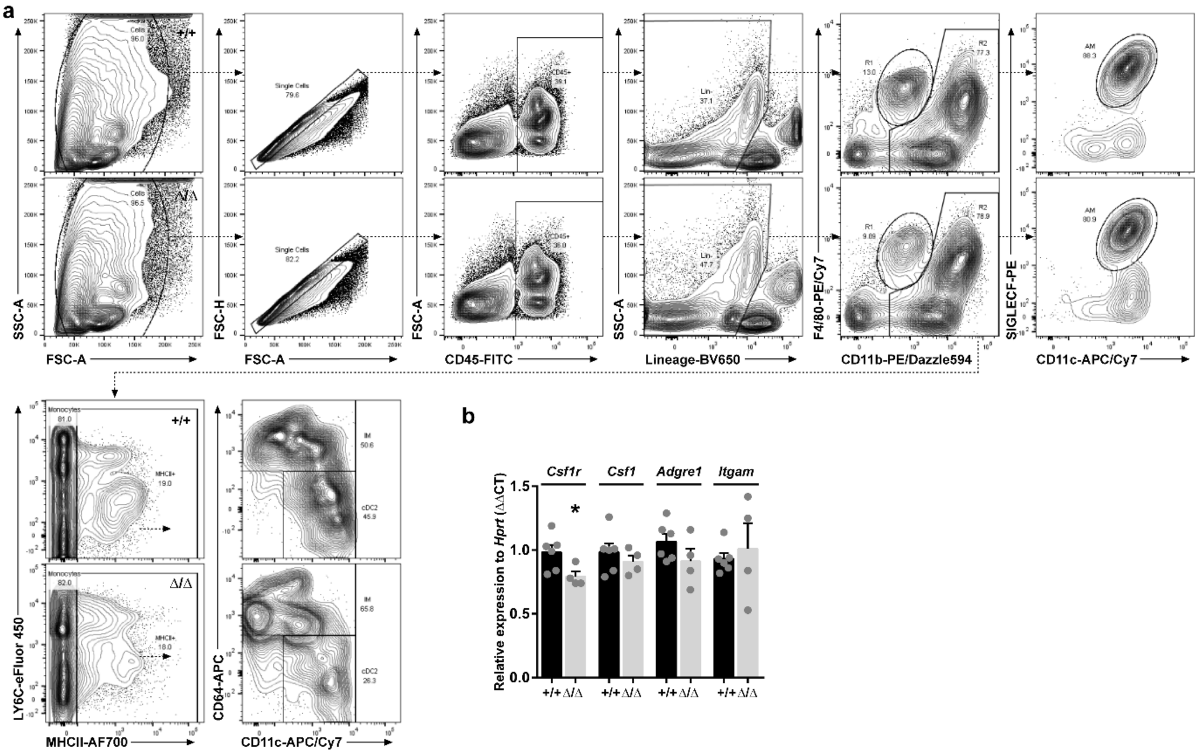

Supplementary Figure 5 – Deletion of FIRE had no effect on macrophage populations in the lung.

a, Single cell suspensions of digested lungs from mice aged between 8-10 weeks were analyzed by flow cytometry as shown in Fig.4c. Alveolar macrophages (AM) were gated as CD45^{+}, lineage^{-} (Lin^{-}; CD3^{-}CD19^{-}LY6G^{-}) and F4/80^{+}CD11b^{lo}CD11c^{+}SIGLECF^{+} (Panels 3 - 6). The CD11b^{+}F4/80$^{-/+}$ cells (R2) were further analyzed to define the monocytes (Mo, MHCII^{-} Ly6C$^{-/+}$, Panel 7), interstitial macrophages (IM, MHCII^{+}CD11c$^{-/+}$CD64^{+}) and conventional dendritic cells subset 2 (cDC2, MHCII^{+}CD11c^{+}CD64^{-}) in Panel 8. **b,** Total RNA was isolated from whole lungs of mice aged 6 weeks. cDNA was prepared and analyzed by qPCR for gene expression. Graph shows mean + SEM. n = 6 +/+ and 4 Δ/Δ. *P* = 0.041 (*) via a two-tailed t-test**.** All source data are provided within a Source Data excel file.

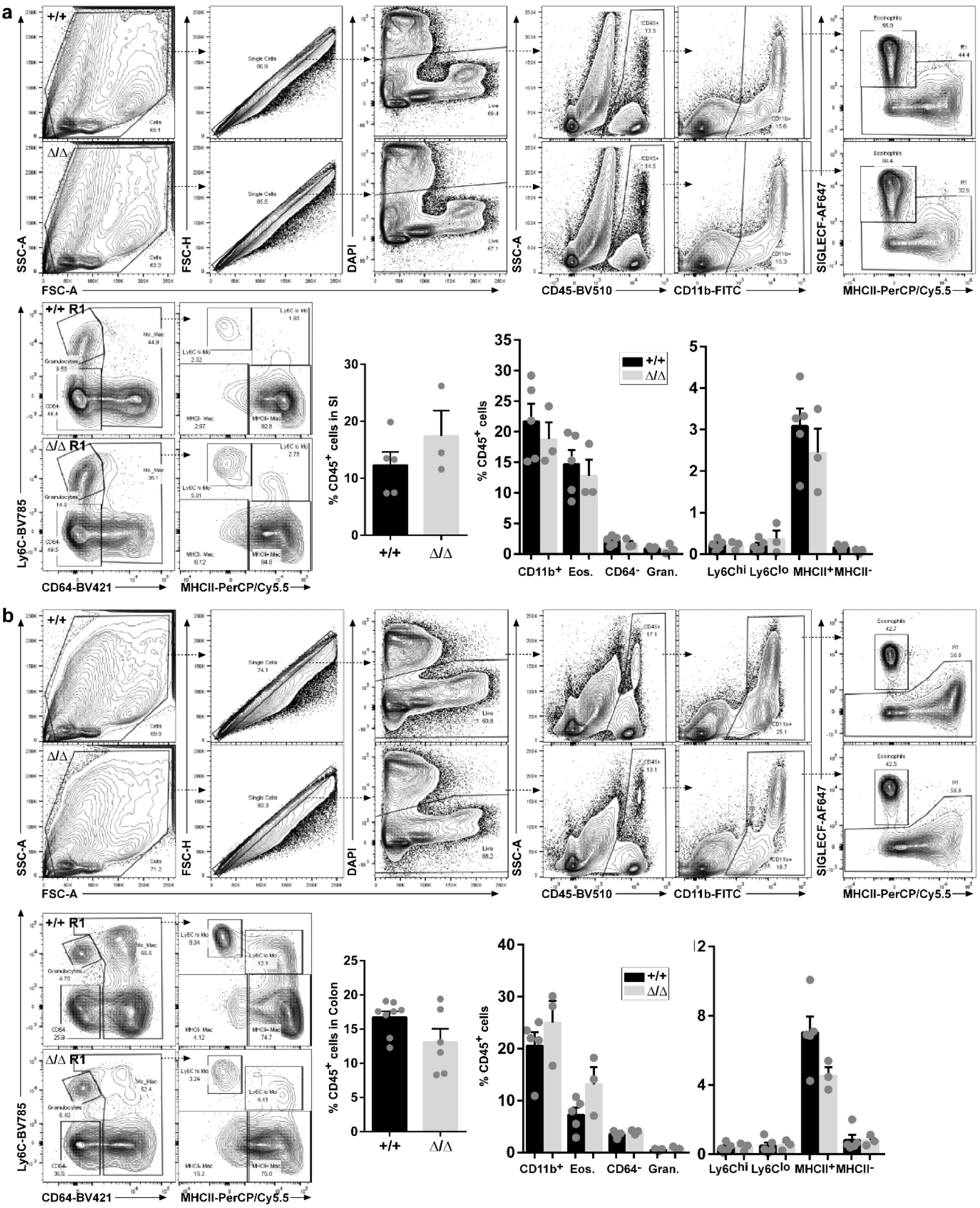

Supplementary Figure 6 – Deletion of FIRE had no effect on macrophage populations in the intestines.

Single-cell suspensions of digested small (**a**) and large (**b**) intestines from mice aged between 7-11 weeks were analyzed by flow cytometry. Cell populations were gated as $CD45^{+}CD11b^{+}$. Dead cells were excluded with DAPI. Eosinophils were gated as $SIGLECF^{+}MHCII^{-}$ (Panel 6). The $SIGLECF^{-}MHCII^{+/-}$ cells (R1) were further analyzed to define the granulocytes

($Ly6C^+CD64^-$) and $Ly6C^-CD64^-$ macrophages (Panel 7). $Ly6C^+CD64^+$ cells (Mo_mac) were further analyzed to identify $Ly6C^{hi/low}$ monocytes and $MHCII^{+/-}$ macrophages (Panel 8). Graphs show mean + SEM. n = 8 +/+ and 6 Δ/Δ for % $CD45^+$ cells in colon. n = 5 +/+ and 3 Δ/Δ for all other cell types in both tissues. All $P > 0.0827$ via a two-tailed t-test. All source data are provided within a Source Data excel file.

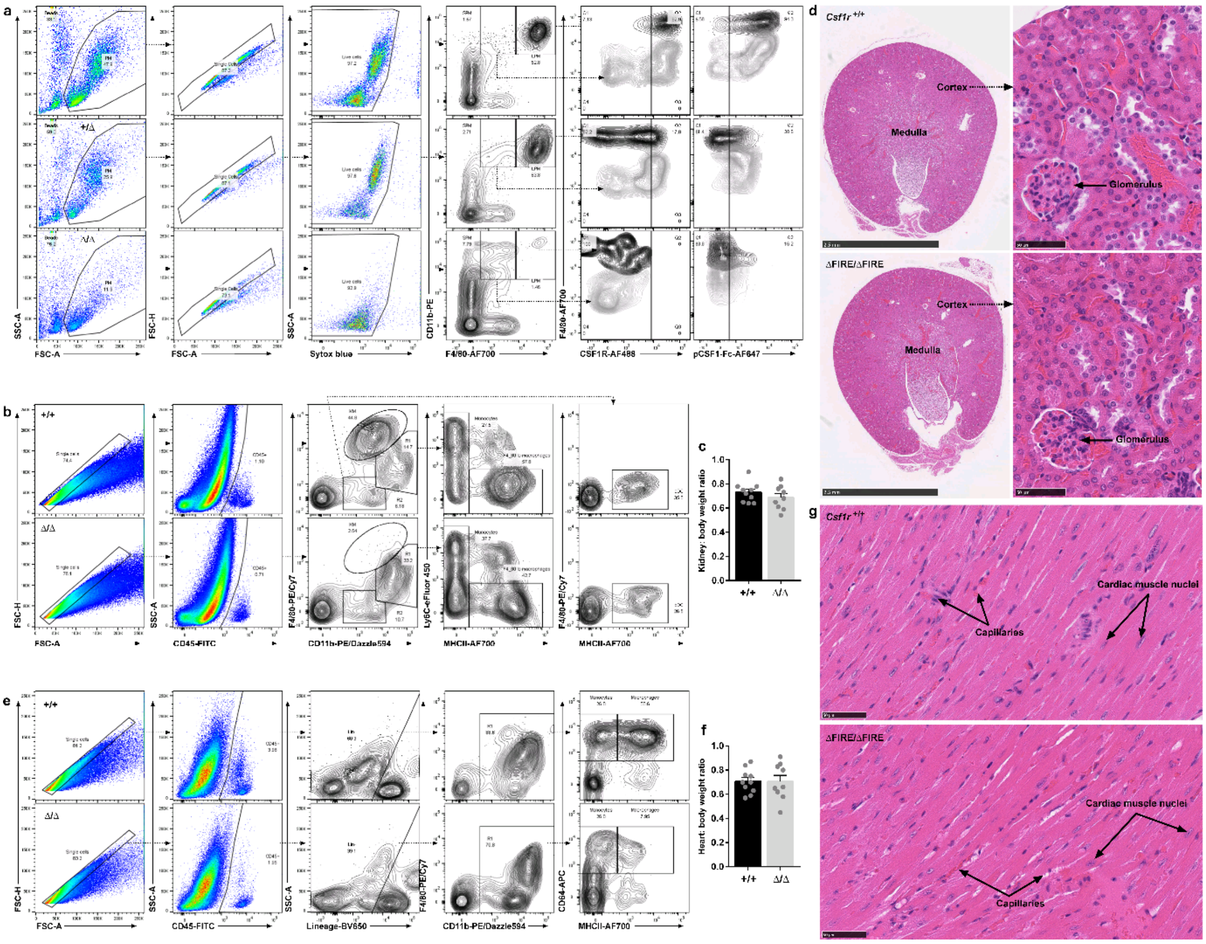

Supplementary Figure 7 – Flow cytometry gating strategies for peritoneal cavity, heart and kidney.

a, Flow cytometry profiles show peritoneal cells from representative *Csf1r*$^{+/+}$ and *Csf1r*$^{\Delta FIRE/\Delta FIRE}$ mice using the gating strategy described in [3] and shown in Fig.5a. Small peritoneal macrophages (SPM) were identified as F4/80^{lo}CD11b^{+} and large peritoneal macrophages (LPM) as F4/80^{hi}CD11b^{+} (Panel 4). These populations were analyzed for CSF1R expression (Panel 5) and binding of pCSF1-Fc^{AF647} (Panel 6). **b,** Leukocyte populations were isolated from enzymatically digested kidneys and gated for CD45 expression. The flow cytometry profiles show analysis of representative *Csf1r*$^{+/+}$ and *Csf1r*$^{\Delta FIRE/\Delta FIRE}$ mice as shown in Fig.5b. Kidney macrophages (KM) were identified as F4/80^{hi}CD11b^{lo} (Panel 3), pooled LY6C$^{-/+}$ monocytes (Mo) as F4/80^{lo}MHCII^{-} (Panel 4), putative monocyte-derived macrophages as F4/80^{lo}MHCII^{+} (Panel 4) and conventional dendritic cells (cDC) as F4/80^{-}CD11b^{lo}MHCII^{+} (Panel 5). **c,** Kidney to body weight ratios were determined from mice aged between 7 – 12 weeks. The average weight of both kidneys was used. n = 10 +/+ and 9 Δ/Δ. **d,** Formalin-fixed and paraffin-embedded (FFPE) kidneys from 7 – 11 week old mice were stained with Hematoxylin and Eosin (H&E). Images are representative of 6 mice per genotype. Scale bars = 2.5 mm (whole kidney) and 50 µm (cortex). **e,** Single-cell

suspensions of enzymatically digested hearts were analyzed by flow cytometry as shown in Fig.5c. Cells were gated lineage$^-$ (Lin$^-$ = CD3/CD19/LY6G). **f,** Heart to body weight ratios were determined from mice aged between 7 – 12 weeks. n = 10 +/+ and 9 Δ/Δ. **g,** FFPE hearts from 7 – 11 week old mice were stained with H&E. Images are representative of 6 mice per genotype. Scale bar = 50 µm. All source data are provided within a Source Data excel file. Graphs show mean + SEM.

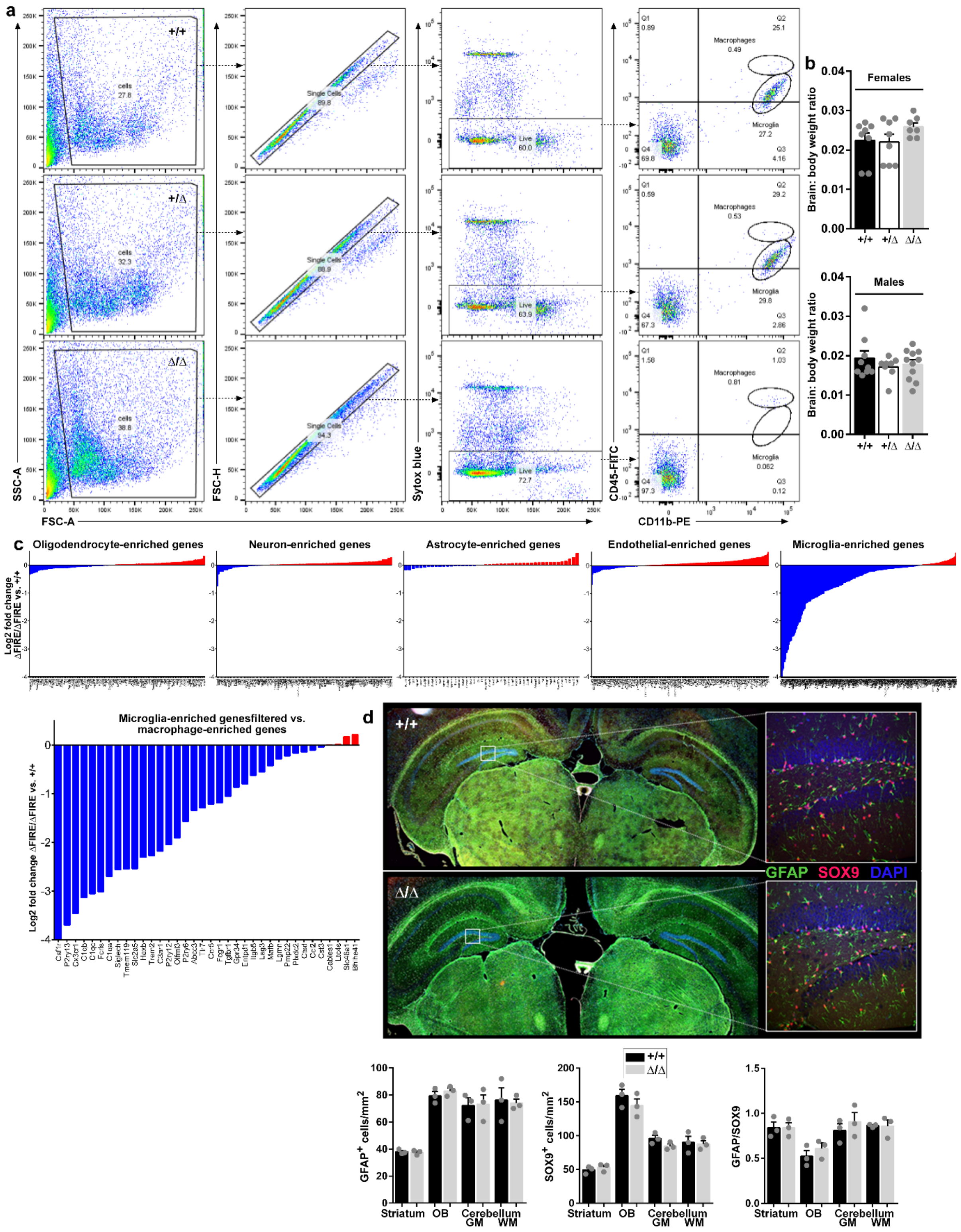

Supplementary Figure 8 – Further analysis of *Csf1r* $^{\Delta FIRE/\Delta FIRE}$ brains

a, Single-cell suspensions of myelin-depleted brains from males and females aged 10 weeks to 9 months were analyzed by flow cytometry for CD45 and CD11b expression as per [4] and shown in Fig.7c. Microglia = CD45^{low}CD11b^{+}. Macrophages = CD45^{+}CD11b^{+}. **b,** Brain to body weight ratios were determined from mice aged 4 – 14 weeks. n = 8, 8, 7 (females) and 9, 8,

11 (males) for +/+, +/Δ and Δ/Δ, respectively. **d,** RNA was extracted from hippocampi from wild type and *Csf1r*$^{\Delta FIRE/\Delta FIRE}$ mice and subjected to microarray-based transcriptome analysis. These data were interrogated with sets of genes whose expression is highly enriched in specific cell types of the brain (astrocytes, oligodendrocytes, microglia, neurons, brain endothelial cells). For each gene, in each cell type-enriched set, the log(2) fold difference in expression in *Csf1r*$^{\Delta FIRE/\Delta FIRE}$ hippocampi, relative to wild type, is shown in numerical order, starting at the lowest log(2) fold difference. For gene sets of enriched oligodendrocyte, neuron, astrocyte, and endothelial genes, very little perturbations were observed. In contrast, a strong down-regulation of microglial-enriched genes was observed. The microglia-enriched gene set was further filtered by selecting only genes uniquely or highly expressed in microglia versus macrophage, myeloid and other immune cells, as defined by Butovsky *et al.*[5] which, when used to interrogate the *Csf1r*$^{\Delta FIRE/\Delta FIRE}$ microarray data, revealed a down-regulation even stronger than that of the unfiltered microglia-enriched genes. The cell-associated gene lists can be found in Supplementary Data 2. **e,** Formalin-fixed and paraffin-embedded adult brains were stained with antibodies against GFAP and SOX9 and the number of GFAP^{+} and SOX9^{+} astrocytes were determined in striatum, olfactory bulb (OB), cerebellar grey matter (GM) and white matter (WM). n = 3 per genotype. All source data are provided within a Source Data excel file. Graphs show mean + SEM.

Supplementary Tables

Supplementary Table 1– Oligonucleotides used for sequencing FIRE deletions

Oligo	Code	Sequence
Upstream FIRE - F1:	Seq-A	5' GCTGCCCTGTCACTGTGTA
Upstream FIRE - F2:	Seq-B	5' CTGTCACTGTGTAGGAAGGG
Inside FIRE - F1:	Seq-C	5' CAACGAGCCTCCTTCCCTAA
Inside FIRE - R1:	Seq-D	5' GAAAGGAGAACTCAAACCCC
Downstream FIRE - R1:	Seq-E	5' CTTCCAAGTGTCTCCTGCTG
Downstream FIRE - R2:	Seq-F	5' TCGTTTCCCATCCCAGGA

F#: forward oligo; R#: reverse oligo.

Supplementary Table 2– Flow cytometry antibodies

Marker	Antibody	Clone	Conjugate	Dilution	Tissue	Supplier	Catalogue #
**ADGRE1 (F4/80)**	Rat IgG2a	BM8	AF488	1 : 500	Blood	BioLegend	123120
				1 : 400	BMDM		
			AF700	1 : 400	Spleen	BioLegend	123130
				1 : 500	PM, BM		
			APC	1 : 800	RAW 264.7	BioLegend	123116
			PE/Cy7	1 : 200	Liver, lung, kidney, heart	BioLegend	123113
**B220**	Rat IgG2a	RA3-6B2	BV510	1 : 800	Blood, BM	BD	563103
				1 : 600	Spleen		
**CD11b**	Rat IgG2b	M1/70	PE	1 : 5000	Blood	BioLegend	101207
				1 : 7000	PM, BMDM		
				1 : 500	Brain		
			PE/Dazzle594	1 : 400	Liver, lung, kidney, heart	BioLegend	101256
			PE/Cy5	1 : 1000	Spleen	BioLegend	101210
			FITC	1 : 7000	Intestines	BioLegend	101205
**CD11c**	Armenian Hamster IgG	N418	APC/Cy7	1 : 200	Lung	BioLegend	117324
		HL3	FITC	1 : 200	Spleen	BD	553801
**CD45**	Rat IgG2b	30-F11	FITC	1 : 500	Brain	BioLegend	103107
			Pacific Blue	1 : 1600	BM	BioLegend	103126
			BV510	1 : 500	Intestines	BioLegend	103137
**CD45.2**	Mouse IgG2a	104	FITC	1 : 200	Liver, lung, kidney, heart	BioLegend	109806
**CD64**	Mouse IgG1	X54-5/7.1	APC	1 : 200	Lung, heart	BioLegend	139306
			BV421	1 : 200	Intestines	BioLegend	139309
**CSF1R (CD115)**	Rat IgG2a	AFS98	PE	1 : 200	RAW 264.7	Thermo Fisher Scientific	12-1152-81
				1 : 300	BM		
**KIT (CD117)**	Rat IgG2b	2B8	FITC	1 : 200	BM	BioLegend	105805
**FLT3 (CD135)**	Rat IgG2a	A2F10	APC	1 : 160	BM	BioLegend	135310
**Lineage markers**							
**CD3**	Rat IgG2b	17A2	Biotin + Streptavidin BV650	1 : 200	Liver, lung, heart	BioLegend	100244
**CD19**	Rat IgG2a	6D5		1 : 1000		BioLegend	115504
**LY6G**	Rat IgG2a	1A8				BioLegend	127604
**LY6C**	Rat IgG2a	ER-MP20	AF647	1 : 2000	Blood, BM	BIO-RAD	MCA2389A
		HK1.4	eFluor 450	1 : 200	Liver, lung, kidney	Thermo Fisher Scientific	48-5932-82
			BV785	1 : 200	Intestines	BioLegend	128041
**LY6G**	Rat IgG2a	1A8	PE	1 : 400	Spleen	BioLegend	127607
**MHCII**	Rat IgG2b	M5/114.15.2	AF700	1 : 400	Liver, lung, kidney, heart	BioLegend	107622
			PerCP/Cy5.5	1 : 300	Intestines	BioLegend	107625
**SIGLECF**	Rat LOU IgG2a	E50-2440	PE	1 : 200	Lung	BD	552126
			AF647	1 : 200	Intestines	BD	562680
**TIM4**	Rat IgG2a	RMT4-54	PE	1 : 200	Liver	BioLegend	130006

Supplementary Table 3– Primers used in qRT-PCR

Gene Symbol	Gene Name	NCBI Reference Sequence		Oligo sequence	Location (exon)	Bp
Adgre1[1]	Adhesion G protein-coupled receptor E1	NM_010130.4	F:	5' TCTGGGGAGCTTACGATGGA	15-16	103
			R:	5' ACAGCAGGAAGGTGGCTATG	16	
Csf1[2]	Macrophage colony-stimulating factor	NM_007778.4	F:	5'AGTATTGCCAAGGAGGTGTCAG	3	107
			R:	5'ATCTGGCATGAAGTCTCCATTT	4	
Csf1r[1]	Macrophage colony-stimulating factor receptor	NM_001037859.2	F:	5' CAGTTCAGAGTGATGTGTGGTC	19	95
			R:	5' CTTGTTGTTCACTAGGATGCCG	20	
Hprt[1]	Hypoxanthine guanine phosphoribosyl transferase	NM_013556.2	F:	5' GCGATGATGAACCAGGTTATGA	2	135
			R:	5' CCTTCATGACATCTCGAGCAAG	3	
Itgam	Integrin alpha M	NM_001082960.1	F:	5' CTGTCACACTGAGCAGAAATCC	26	119
			R:	5' ATTCAGTGTGACCTTCCATCCT	27	

References

1 Kearse, M. *et al.* Geneious Basic: an integrated and extendable desktop software platform for the organization and analysis of sequence data. *Bioinformatics* **28**, 1647-1649 (2012).

2 Hawley, C. A. *et al.* Csf1r-mApple Transgene Expression and Ligand Binding In Vivo Reveal Dynamics of CSF1R Expression within the Mononuclear Phagocyte System. *J. Immunol.* (2018).

3 Bain, C. C. *et al.* Long-lived self-renewing bone marrow-derived macrophages displace embryo-derived cells to inhabit adult serous cavities. *Nature communications* **7**, ncomms11852 (2016).

4 Grabert, K. *et al.* Microglial brain region-dependent diversity and selective regional sensitivities to aging. *Nat. Neurosci.* **19**, 504-516 (2016).

5 Butovsky, O. *et al.* Identification of a unique TGF-beta-dependent molecular and functional signature in microglia. *Nat. Neurosci.* **17**, 131-143 (2014).
