# Supplementary material for: Deletion of a Csf1r enhancer selectively impacts CSF1R expression and development of tissue macrophage populations
Source: Nat Commun. 2019 Jul 19;10:3215. doi: 10.1038/s41467-019-11053-8 (PMC6642117; doi:10.1038/s41467-019-11053-8)
Supplement: Supplementary file 3 — Reporting Summary [file 41467_2019_11053_MOESM3_ESM.pdf]

nature research

Corresponding author(s): Clare Pridans

Last updated by author(s): Jun 12, 2019

Reporting Summary

Nature Research wishes to improve the reproducibility of the work that we publish. This form provides structure for consistency and transparency in reporting. For further information on Nature Research policies, see Authors & Referees and the Editorial Policy Checklist.

Statistics

For all statistical analyses, confirm that the following items are present in the figure legend, table legend, main text, or Methods section.

n/a	Confirmed	
☐	☒	The exact sample size (*n*) for each experimental group/condition, given as a discrete number and unit of measurement
☒	☐	A statement on whether measurements were taken from distinct samples or whether the same sample was measured repeatedly
☐	☒	The statistical test(s) used AND whether they are one- or two-sided<br>*Only common tests should be described solely by name; describe more complex techniques in the Methods section.*
☒	☐	A description of all covariates tested
☒	☐	A description of any assumptions or corrections, such as tests of normality and adjustment for multiple comparisons
☐	☒	A full description of the statistical parameters including central tendency (e.g. means) or other basic estimates (e.g. regression coefficient) AND variation (e.g. standard deviation) or associated estimates of uncertainty (e.g. confidence intervals)
☐	☒	For null hypothesis testing, the test statistic (e.g. F, t, r) with confidence intervals, effect sizes, degrees of freedom and P value noted<br>*Give P values as exact values whenever suitable.*
☒	☐	For Bayesian analysis, information on the choice of priors and Markov chain Monte Carlo settings
☒	☐	For hierarchical and complex designs, identification of the appropriate level for tests and full reporting of outcomes
☒	☐	Estimates of effect sizes (e.g. Cohen's d, Pearson's r), indicating how they were calculated

Our web collection on statistics for biologists contains articles on many of the points above.

Software and code

Policy information about availability of computer code

Data collection	No software was used.
Data analysis	FlowJo v10 was used for flow cytometry analysis.<br>Graphpad Prism 6 was used to generate bar graphs and perform statistical analysis.<br>Affymetrix Transcription Analysis Console was used to analyze microarray data.

For manuscripts utilizing custom algorithms or software that are central to the research but not yet described in published literature, software must be made available to editors/reviewers. We strongly encourage code deposition in a community repository (e.g. GitHub). See the Nature Research guidelines for submitting code & software for further information.

Data

Policy information about availability of data

All manuscripts must include a data availability statement. This statement should provide the following information, where applicable:

- Accession codes, unique identifiers, or web links for publicly available datasets
- A list of figures that have associated raw data
- A description of any restrictions on data availability

A full data availability statement is included in the manuscript.

Field-specific reporting

Please select the one below that is the best fit for your research. If you are not sure, read the appropriate sections before making your selection.

☒ Life sciences ☐ Behavioural & social sciences ☐ Ecological, evolutionary & environmental sciences

For a reference copy of the document with all sections, see nature.com/documents/nr-reporting-summary-flat.pdf

Life sciences study design

All studies must disclose on these points even when the disclosure is negative.

Sample size	In determining the sample size, we are asking the question: is the macrophage population of interest there or not in the FIRE deficient mice? We have determined the sample size based on data from our analysis of Csf1r deficient rats (PMID: 30249809). A sample size of 2 would ensure a power of 0.9998752 with 5% error (calculated in G power). Our sample sizes however, are all > 2.
Data exclusions	No data has been excluded.
Replication	All of our analyses have successfully been reproduced within our laboratory by different members. To achieve this, we have regulated 'standard operating procedures' for each protocol, maintained by senior staff. In addition, some analyses were performed by collaborators in different countries.
Randomization	Our study did not include experimental groups.
Blinding	The majority of histological samples were blinded during image acquisition.

Reporting for specific materials, systems and methods

We require information from authors about some types of materials, experimental systems and methods used in many studies. Here, indicate whether each material, system or method listed is relevant to your study. If you are not sure if a list item applies to your research, read the appropriate section before selecting a response.

Materials & experimental systems

n/a	Involved in the study	
☐	☒	Antibodies
☐	☒	Eukaryotic cell lines
☒	☐	Palaeontology
☐	☒	Animals and other organisms
☒	☐	Human research participants
☒	☐	Clinical data

Methods

n/a	Involved in the study	
☒	☐	ChIP-seq
☐	☒	Flow cytometry
☒	☐	MRI-based neuroimaging

Antibodies

Antibodies used	Details of all antibodies are included within the text or within Supplementary Table 2.
Validation	All antibodies had been previously validated (and published) in other mouse studies within ours and others laboratories.

Eukaryotic cell lines

Policy information about cell lines

Cell line source(s)	ATCC
Authentication	RAW264.7 cells are very distinct in morphology and have been tested for expression of known markers (Csf1r, F4/80).<br>E14 cells have been used to generate transgenic mice within our facility, can tri-lineage differentiate and also differentiate into macrophages.
Mycoplasma contamination	Cells have been tested for mycoplasma.
Commonly misidentified lines<br>(See ICLAC register)	No commonly misidentified cell lines were used.

Animals and other organisms

Policy information about studies involving animals; ARRIVE guidelines recommended for reporting animal research

Laboratory animals	Mouse. Ages specified in the manuscript. All analyses performed on male and female mice. B6CBAF1/J x C57BL/6 background.
Wild animals	Our study did not involve wild animals.
Field-collected samples	Our study did not involve samples collected from the field.
Ethics oversight	This study was performed under authority of a UK Home Office License and approved by the University of Edinburgh Animal Welfare and Ethical Review Body (AWERB).

Note that full information on the approval of the study protocol must also be provided in the manuscript.

Flow Cytometry

Plots

Confirm that:

☒ The axis labels state the marker and fluorochrome used (e.g. CD4-FITC).

☒ The axis scales are clearly visible. Include numbers along axes only for bottom left plot of group (a 'group' is an analysis of identical markers).

☒ All plots are contour plots with outliers or pseudocolor plots.

☒ A numerical value for number of cells or percentage (with statistics) is provided.

Methodology

Sample preparation	Sample preparation is described in the manuscript.
Instrument	BD LSR Fortessa
Software	BD software to collect data, FlowJo v10 to analyze data.
Cell population abundance	We enriched the GFP+ populations after transfection and plated single GFP+ cells. Details can be found in the Source Data file.
Gating strategy	The full gating strategy for all tissues is provided within the main text or Supplementary Information. Gates and quadrants were determined with isotype controls or FMOs for all experiments.

☒ Tick this box to confirm that a figure exemplifying the gating strategy is provided in the Supplementary Information.
