# Supplementary material for: Deletion of a Csf1r enhancer selectively impacts CSF1R expression and development of tissue macrophage populations
Source: Nat Commun. 2019 Jul 19;10:3215. doi: 10.1038/s41467-019-11053-8 (PMC6642117; doi:10.1038/s41467-019-11053-8)
Supplement: Supplementary file 4 — Description of Additional Supplementary Files [file 41467_2019_11053_MOESM4_ESM.pdf]

Description of Additional Supplementary Files

Supplementary Data 1

Microarray analysis of intestines from wild type and FIRE deficient mice.

Supplementary Data 2

Microarray analysis of brain from wild type and FIRE deficient mice.
